# Detection of Peramivir and Laninamivir, New Anti-Influenza Drugs, in Sewage Effluent and River Waters in Japan

**DOI:** 10.1371/journal.pone.0131412

**Published:** 2015-06-25

**Authors:** Takashi Azuma, Hirotaka Ishiuchi, Tomomi Inoyama, Yusuke Teranishi, Misato Yamaoka, Takaji Sato, Naoyuki Yamashita, Hiroaki Tanaka, Yoshiki Mino

**Affiliations:** 1 Osaka University of Pharmaceutical Sciences, Takatsuki, Osaka, Japan; 2 Research Center for Environmental Quality Management, Kyoto University, Otsu, Shiga, Japan; NSYSU, TAIWAN

## Abstract

This is the first report of the detection of two new anti-influenza drugs, peramivir (PER) and laninamivir (LAN), in Japanese sewage effluent and river waters. Over about 1 year from October 2013 to July 2014, including the influenza prevalence season in January and February 2014, we monitored for five anti-influenza drugs—oseltamivir (OS), oseltamivir carboxylate (OC), zanamivir (ZAN), PER, and LAN—in river waters and in sewage effluent flowing into urban rivers of the Yodo River system in Japan. The dynamic profiles of these anti-influenza drugs were synchronized well with that of the numbers of influenza patients treated with the drugs. The highest levels in sewage effluents and river waters were, respectively, 82 and 41 ng/L (OS), 347 and 125 ng/L (OC), 110 and 35 ng/L (ZAN), 64 and 11 ng/L (PER), and 21 and 9 ng/L (LAN). However, application of ozone treatment before discharge from sewage treatment plants was effective in reducing the levels of these anti-influenza drugs in effluent. The effectiveness of the ozone treatment and the drug dependent difference in susceptibility against ozone were further evidenced by ozonation of a STP effluent in a batch reactor. These findings should help to promote further environmental risk assessment of the generation of drug-resistant influenza viruses in aquatic environments.

## Introduction

In recent years, a new environmental pollution problem has been reported whereby residual components of anti-influenza drugs are detected in the river water environment [1,2]. Research has been done into Tamiflu (oseltamivir: OS) and Relenza (zanamivir: ZAN), which are drugs used globally to treat annual seasonal influenza and could be effective in alleviating the potential damage from future global pandemics of new strains of influenza viruses [3–6]. There is concern that oseltamivir carboxylate (OC), which is the pharmacologically active metabolite of OS, could carry environmental risks in terms of increasing the development of drug-resistant viruses in wild waterfowl inhabiting river environments; these resistant viruses could then propagate among humans [7–9]. For this reason, research that aims to determine the status of OS and ZAN in sewage effluent and river waters [5,6,10,11], as well as the behavior of these drugs in river environments [12–17], is beginning to be actively pursued.

In Japan, two new anti-influenza drugs were introduced clinically in 2010. One of them was Rapiacta (peramivir: PER), which is administered by intravenous drip injection [18–20], and the other is Inavir (laninamivir octanoate), which is administered by inhalation in a way similar to Relenza [19,21,22]. The mechanism of pharmacological action of these two drugs is, similar to that of Tamiflu and Relenza, to inhibit the neuraminidase glycoproteins involved in the proliferation of influenza viruses and suppress the proliferation of these viruses in the human body [19]. Additionally, although Inavir, similarly to Tamiflu, is in an inactive form when taken, it is a prodrug, meaning that its pharmacologically active metabolite, laninamivir (LAN), has medical effects [19,21,22].

Japan accounts for more than 70% of the world’s Tamiflu consumption and compared with other countries is extremely highly dependent on anti-influenza drugs [23]. For this reason, it is probable that along with the clinical introduction of the new drugs have come new water environment pollution problems additional to those created by Tamiflu and Relenza [5,6,10,11]. However, to our knowledge there has still been no report of the status of these two new drugs in sewage effluent and river waters, and there are still many factors that need to be revealed before an environmental risk assessment can be conducted. Therefore, it is important to gain an understanding of the status of Rapiacta and Inavir, in addition to Tamiflu and Relenza, in the river environment.

Here, we clarified the status of the new drugs Rapiacta and Inavir in sewage effluent and river waters by performing a year-round detailed monitoring survey of the urban rivers flowing into a representative river basin in Japan, the Yodo River basin, from 2013 to 2014, centering on the height of the influenza outbreak season. In addition, with the aim of finding a water treatment technique effective in removing these drugs, we ran a laboratory-scale removal experiment using ozonation, which is reportedly effective in removing various components of pharmaceutical and personal care products [24–27].

## Materials and Methods

### 2.1 Chemicals and reagents

OS (purity 99%) and ZAN (purity 99%) were purchased from LKT Laboratories (St. Paul, MN, USA). LAN (purity 98%) was purchased from Toronto Research Chemicals Inc. (Toronto, Ontario, Canada). OC (purity 99%) was purchased from Acanthus Research Inc. (Mississauga, Ontario, Canada). PER (purity 99%) was provided by Shionogi & Co., Ltd. (Osaka, Japan) and purchased from ChemScene, LLC. (Monmouth Junction, NJ, USA). The chemical structures and physicochemical properties of these anti-influenza drugs [22,28–37] are shown in Table 1. It is noted in similarities of chemical and physicochemical properties of ZAN and LAN. LC-MS-grade solvents (methanol, acetone, and acetonitrile), formic acid, hydrochloric acid, ascorbic acid, and triethylamine were purchased from Wako Pure Chemical Industries, Ltd. (Osaka, Japan). Individual standard stock solutions of OS, OC, ZAN, PER, and LAN at 1 mg/100 mL were prepared in methanol and stored at –30°C.

**Table 1:** Chemical structures and physicochemical properties of the anti-influenza drugs examined [22, 28–37].

Compound	Molecular formula	Molecular mass (g/mol)	p*K*_a_	Log*P*	Excreation rate (%)
Oseltamivir (OS)	C_16_H_28_N_2_O_4_	312.41	7.7 [30,31]	0.4 [31]	15 [28]
Oseltamivir carboxylate (OC)	C_14_H_24_N_2_O_4_	284.35	3.8 (acid) [32,37], 7.8 (base) [32,37]	-2.1 [34]	80 [28]
Zanamivir (ZAN)	C_12_H_20_N_4_O_7_	332.31	3.8 (acid) [37], 11.3 (base) [37]	-7.1 [35]	80 [29]
Peramivir (PER)	C_15_H_28_N_4_O_4_	328.41	4.1 (acid) [33], 13.1 (base) [33]	-1.4 [33]	91 [36]
Laninamivir (LAN)	C_13_H_22_N_4_O_7_	346.34	3.8 (acid) [33], 11.3 (base) [33]	-3.1 [33]	13 [22]

### 2.2 Sampling sites

**Fig 1:**
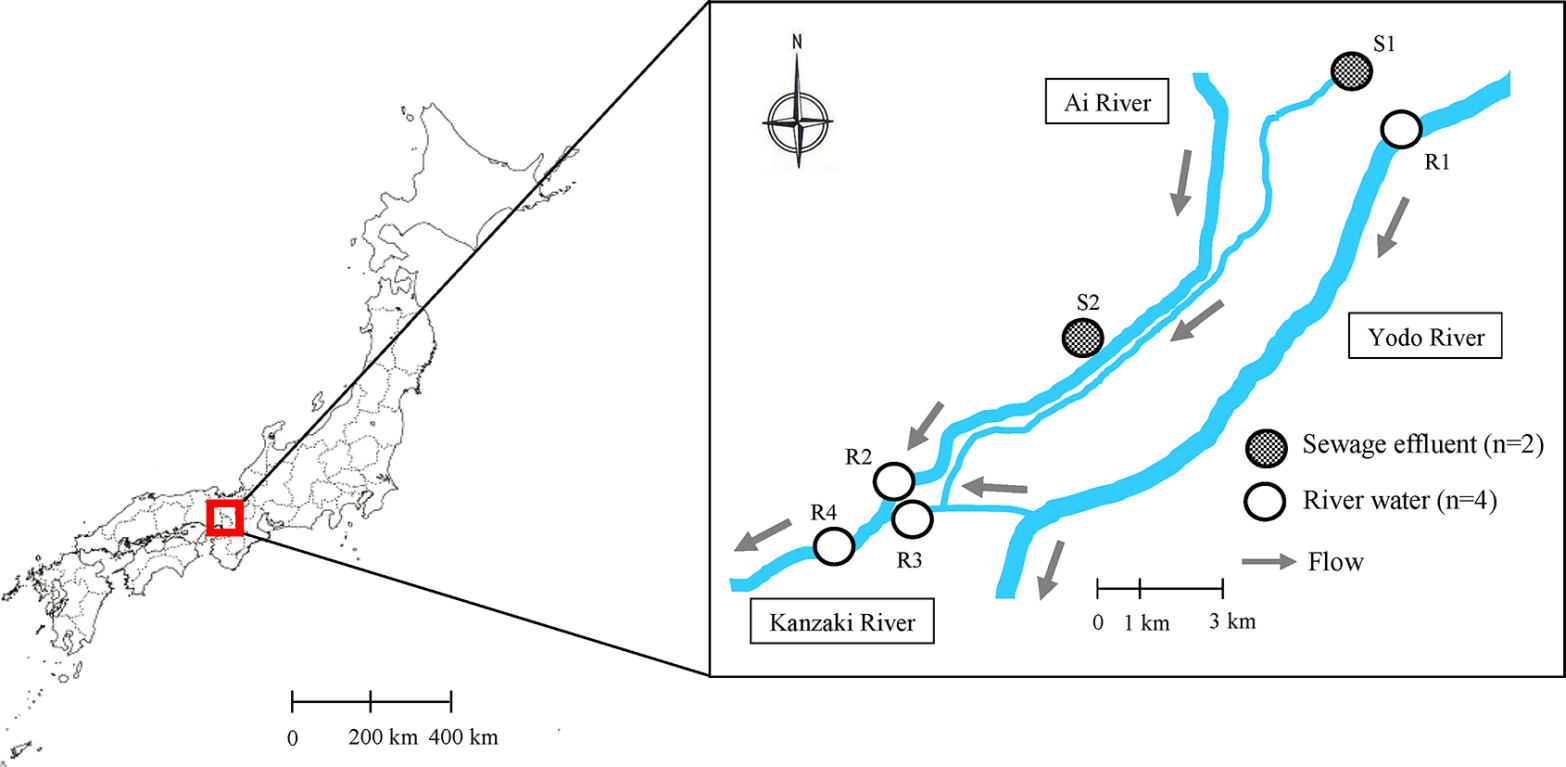
Locations of sampling sites in the Yodo River system.

River waters were collected at four sites on the middle reaches and downstream areas of the Yodo River basin. The locations were R1 (N 34°48'52.56" E 135°37'56.96"), R2 (N 34°45'30.41" E 135°31'54.90"), R3 (N 34°45'22.35" E 135°32'12.25"), and R4 (N 34°45'07.48" E 135°31'30.50"). The basin is located in the mid-Kansai district; it covers 208 km^2^ and is home to 2 million people [38]. We also sampled effluents from public water boundary regions at two sewage treatment plants (STPs) in the basin, namely S1 (N 34°49'26.93" E 135°37'21.21") and S2 (N 34°46'46.29" E 135°34'14.89"). Both STPs used a conventional activated sludge system (CAS) followed by chlorination for disinfection, and one STP (S2) partly used CAS followed by ozonation (8.6 mg ozone/L). No special permission was required for every sampling location because all waters sampled were of public water body. Locations of the sampling sites are given in Fig 1. Adequateness of the sampling sites is based on the evidence that the STP effluents are the major source of the anti-influenza drug load in the Yodo River system (68–94% of total mass fluxes) due to the high rate of equipment of the sewerage systems in the same area (97–99% in 2010) [16].

Sampling was done over about 1 year from October 2013 to July 2014, including the influenza outbreak season from January to February 2014 on non-rainy days once a week during the influenza outbreak season and once every 2 months for the rest of the year. No rainfall greater than 1 mm was observed in the sampling area during the 2 days before each sampling day. The frequency of the sampling in the present study is the same as that we have done in a year during July 2010 to June 2011 at the similar sites of the Yodo River basin [6] to show synchronous dynamics of observed and predicted values of anti-influenza drugs, OS, OC and ZAN, in environmental waters during a seasonal influenza outbreak in 2011 (January to March). Samples (200 mL) were collected in glass bottles each containing ascorbic acid (1 g/L) as a preservative to keep the sampled solution under reductive state at pH about 3 [39], kept in the refrigerator under dark at 4°C, and processed within 24 h.

### 2.3 Analytical procedures

Water samples were analyzed by using a combination of a strong-cation solid-phase extraction cartridges (Bond Elut SCX, 500 mg; Agilent Technologies, Santa Clara, CA) and liquid chromatography–tandem mass spectrometry (LC-MS/MS) after filtration through a glass fiber filter (GF/B, 1-μm pore size, Whatman, Maidstone, UK) [40]. Only filtered water samples were used for the analysis, because the adsorption of these drugs onto particulate matter in sewage and river water is negligibly small [7,41]. Aliquots (30 mL for river water and 10 mL for STP effluent) were loaded onto the extraction cartridges.

The materials adsorbed onto the cartridges were eluted with 10% triethylamine in a 1:1 (v/v) mixture of acetone and water after the cartridge had been cleaned up with 6 mL MeOH containing 2% formic acid. Each eluate was concentrated under a gentle stream of nitrogen gas. The residue was then re-dissolved in 200 μL of a 9:1 (v/v) mixture of aqueous 0.1% formic acid solution and methanol, and 10 μL of this solution was subjected to LC-MS/MS analysis in a Waters Acquity Ultra Performance LC (UPLC) device fitted with a column (2.1 mm × 100 mm, 1.7 μm) of UPLC BEH C_18_ (Waters Corp., Milford, MA, USA).

A gradient elution program was achieved at 60°C with a mixed solvent system of 0.1% formic acid (v/v) in water (A) and acetonitrile (B) at a flow rate of 0.35 mL/min under a program of 0.0 to 4.0 min (5% B), 4.0 to 4.3 min (25% B), 4.3 to 5.8 min (80% B), 5.8 to 6.0 min (80% B), and 6.0 to 8.0 min (5% B) to condition the column. The UPLC system was coupled to a tandem-quadrupole-detector mass spectrometer (Waters Corp.) equipped with an electrospray ionization source and interface, and it was operated in positive ion mode. Product ions were generated with collision energies of 10 (OS), 10 (OC), 17 (ZAN), 18 (PER), and 16 (LAN) eV. Instrument control and data acquisition and quantification were performed with Mass Lynx 4.1 software (Waters Corp.).

The concentrations of OS, OC, ZAN, PER, and LAN were determined by subtracting the blank data from the data given by the addition of each spiked compound (20 ng/L) to account for matrix effects and loss during sample extraction [5,39,42].

Changes in the numbers of influenza patients per Prefecture per week from October 2013 to July 2014 in Japan were examined by using the surveillance data collected by the National Institute of Infectious Diseases, Japan [43] and the corresponding values in the northern area of Osaka Prefecture including the sampling sites [44].

### 2.4 Experimental removal of anti-influenza drugs by ozonation

To verify the effectiveness of ozone treatment in removing anti-influenza drugs and obtain kinetic insight of the treatment which will further show drug dependent difference in degradability, as the test water we used secondary effluent from an STP before chlorination for disinfection. Sampling was done on 5 February 2014, at the peak of an influenza outbreak [43,44].

All experiments were performed in a cylindrical stainless-steel reactor with an inside diameter of 23 cm, a height of 40.7 cm, and an effective volume of 1.7 L. Details of the reactor and it’s operation have been reported previously [45]. The temperature of the test water was maintained at 20°C by using a water circulator to circulate water of controlled temperature into a water jacket outside the reactor. The test water was stirred continuously with a stirrer in the reactor during the treatment experiments.

The experiments started by sparging ozone gas continuously into the reactor filled with the test water. The time before the start of ozone gas sparging was taken to be 0 min (initial conditions), and 60-mL samples were collected from the reactor 2, 5, 10, 15, and 20 min after the start of the reaction. The ozone feed rate was set to 0.6 mg/L/min on the basis of previous research [24,25] that examined the effectiveness of ozone treatment on a wide range of components used in pharmaceutical and personal care products. The ozone dose in this experiment corresponded to 3 mg/L. Collected samples were analyzed as explained in section 2.3 Analytical procedures.

### 2.5 Method validation

Seven-point calibration curves were constructed for quantification; the concentrations ranged between 0.5 and 500 ng/mL in a 9:1 (v/v) mixture of 0.1% formic acid solution in methanol. Linear calibration curves for OS, OC, ZNA, PER, and LAN were obtained in the concentration range of 0.5 to 500 ng/L (*r* ^2^ > 0.99).

The LOD and LOQ values for the environmental water samples were calculated on the basis of the concentrations at signal to noise ratios of 3:1 and 10:1, respectively [2,42]. Rates of recovery from river waters were in the ranges of 65% ± 4% (OS), 76% ± 5% (OC), 62% ± 9% (ZAN), 76% ± 2% (PER), and 59% ± 2% (LAN); for the STP effluents the rates were 61% ± 9% (OS), 56% ± 3% (OC), 42% ± 5% (ZAN), 70% ± 4% (PER), and 45% ± 2% (LAN). Although the rates of recovery of LAN (also ZAN) were relatively lower than those of OS, OC and PER, it is relevant to estimate the close rates of recovery of LAN (59–45%) and ZAN (62–42%) in which latter values were higher than those (39.2–23.3%) previously published [40] by taking similarity of their chemical and physicochemical properties (Table 1) into consideration. Reproducibility for the river water or STP effluent results was in the ranges of 0.3%–0.7% (OS), 0.4%–0.7% (OC), 0.5%–0.7% (ZAN), 0.5%–1.0% (PER), and 0.3%–0.7% (LAN).

## Results and Discussion

### 3.1 Time-dependent dynamics of OS, OC, ZAN, PER, and LAN in river waters and STP effluent

**Fig 2:**
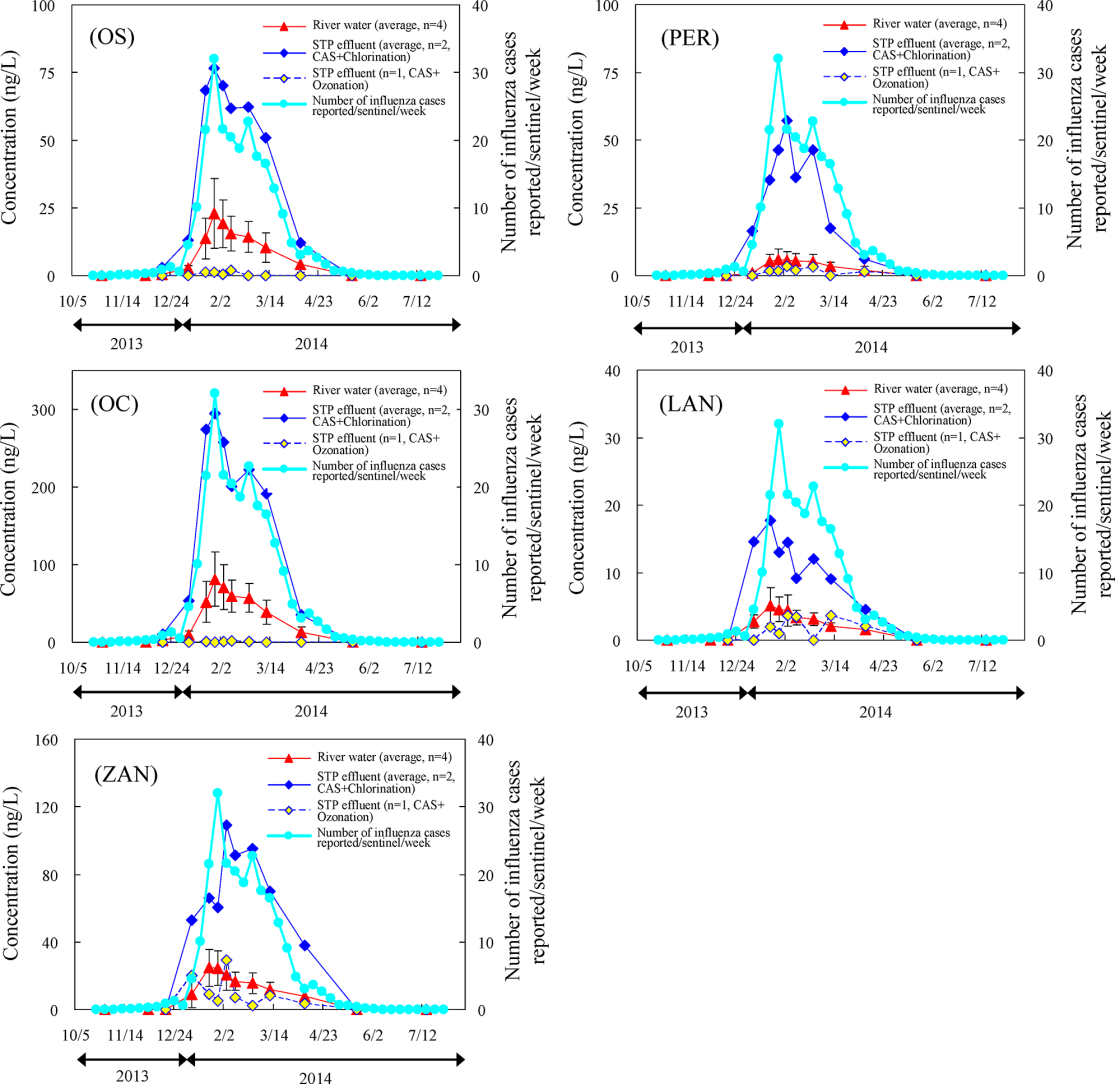
Time-dependent dynamics of OS, OC, ZAN, PER, and LAN average concentrations, with standard deviations, in river waters and STP effluent (CAS: conventional activated sludge systems) from July to June 2013−2014, including in the influenza outbreak season (January to March 2014).

We examined the time-dependent dynamics of OS, OC, ZAN, PER, and LAN concentrations in STP effluent and river waters from July to June 2013−2014, including in the influenza outbreak season (January to March 2014) (Fig 2). We also examined the changes in the numbers of influenza patients (patients/sentinel/week) [44].

OS, OC, ZAN, PER, and LAN were not detected at the end of October 2013. Their levels then began to increase in December by several ng/L synchronously with the increase in the number of influenza patients, peaking at the end of January and in early February 2014. In May 2014, after the end of the outbreak, none of the targeted drugs was detectable. These results were similar to those in a previous report of OS, OC, and ZAN levels in sewage influents and effluents [6].

The maximum concentrations detected in the river water were 10–41 ng/L (OS), 39–125 ng/L (OC), 13–35 ng/L (ZAN), 2–11 ng/L (PER), and 4–9 ng/L (LAN). In the STP effluents they ranged from 74–82 ng/L (OS), 243–347 ng/L (OC), 109–110 ng/L (ZAN), 53–64 ng/L (PER), and 18–21 ng/L (LAN). These results showed that the concentrations of anti-influenza drugs or their metabolites in the STP effluent were roughly two to five times those in the river water; as already indicated in previous surveys of OS, OC, and ZAN [5,16]. Our findings suggested that the STP effluent was a major source of loading of PER and LAN in the river water. Although the levels of PER and LAN were lower than those of OS, OC, and ZAN, it was clear that they were still present in sewage effluent and river water in concentrations from several ng/L to several tens of ng/L during the influenza outbreak season. To our knowledge, this research is the first to report the existence of PER and LAN in sewage effluent and river water. The ratio of OS to OC was in the range of 0.26 ± 0.05 (n = 18) for STP effluent and 0.23 ± 0.05 (n = 36) for river water; these values agreed with those reported in previous research (approximately 0.2–0.3 [5,6,41]). This indicated that the source of the OS and OC detected in the urban rivers studied here was Tamiflu ingested by patients.

The maximum concentrations of the drugs in the effluent from STPs that used the CAS process followed by chlorination for disinfection were 82 ng/L (OS), 347 ng/L (OC), 110 ng/L (ZAN), 64 ng/L (PER), and 21 ng/L (LAN). After ozone treatment, the concentrations of all five components were reduced to several to several tens of ng/L, 2 ng/L (OS), 2 ng/L (OC), 29 ng/L (ZAN), 4 ng/L (PER), and 4 ng/L (LAN). Not only was ozone treatment effective for reducing the levels of OS, OC, and ZAN [2,6] as previously reported—it was also effective in removing the new drug components PER and LAN.

### 3.2 Experimental ozone treatment of OS, OC, ZAN, PER, and LAN in secondary effluent from STP

**Fig 3:**
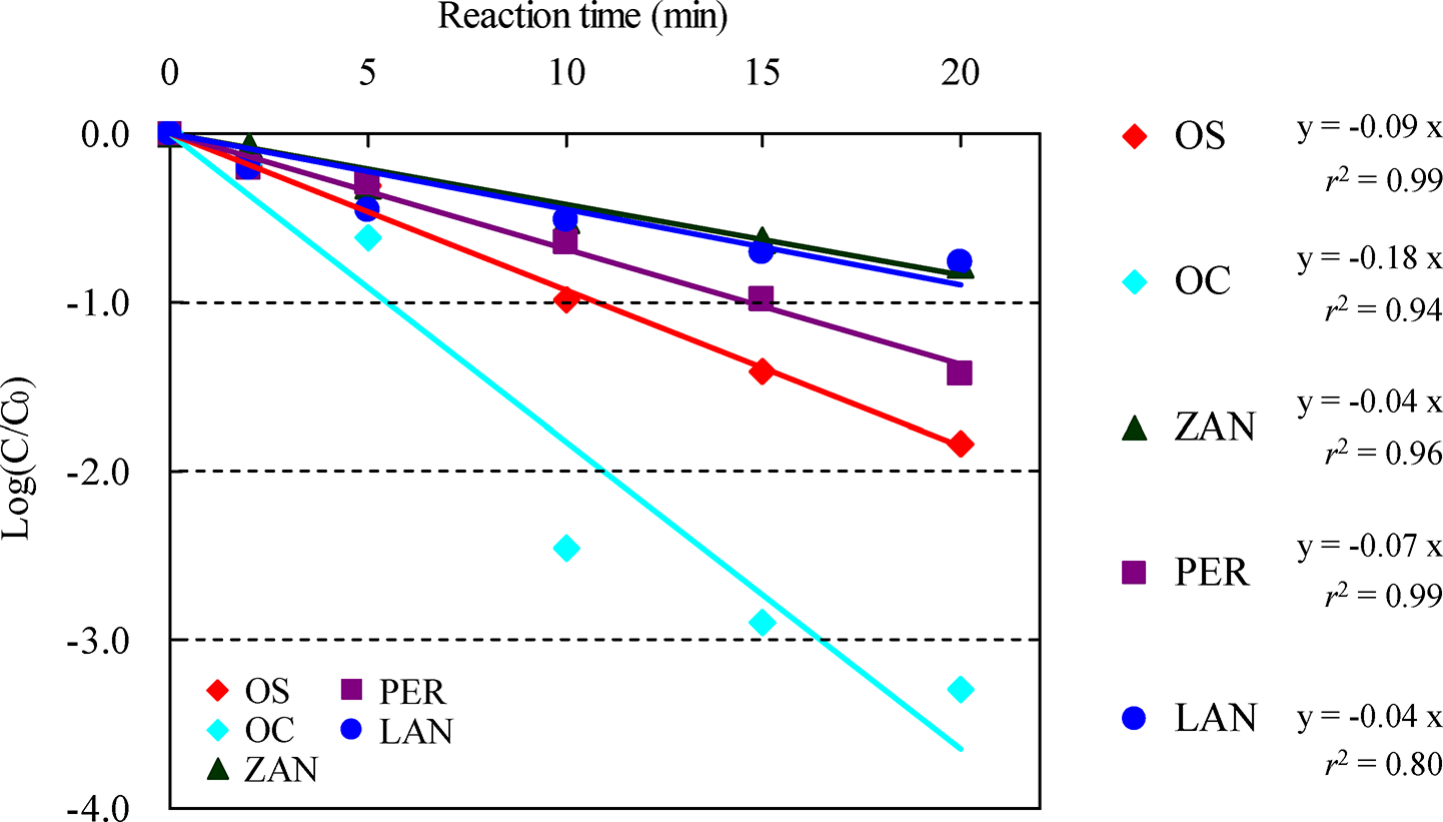
Relative residual concentrations of OS, OC, ZAN, PER, and LAN after experimental ozonation of secondary effluent from an STP at the influenza peak (C_0_: Initial concentration, C: Concentration after reaction).

We also examined the effectiveness of our experimental ozone treatment of OS, OC, ZAN, PER, and LAN in secondary effluent from an STP (Fig 3) by using a batch ozonation reactor. Although the dose of ozone by using this reactor (3 mg/L) is about 1/3 of the practical level treated at the STP (8.6 mg/L), it may be suffice to show the superiority of the treatment. The changes in concentration due to the removal reaction showed good linearity for all components when evaluated on a logarithmic plot of concentration versus reaction time [*r* > 0.99 (OS), 0.97 (OC), 0.98 (ZAN), 0.99 (PER), and 0.95 (LAN); *r* ^2^ > 0.99 (OS), 0.94 (OC), 0.96 (ZAN), 0.99 (PER), and 0.80 (LAN)]. After 20-min reaction, all concentrations became below the detection levels. We consider these profiled values adequate in light of a report stating that ozone treatment of a wide range of components in pharmaceutical and personal care products follows pseudo-first-order kinetics [46]. In addition, the half-lives under these ozone treatment conditions were 3.0 (OS), 1.7 (OC), 7.5 (ZAN), 4.3 (PER), and 7.5 (LAN) min; OC was the most easily removed, followed by OS and PER, and then by ZAN and LAN together. This trend corresponded well to the trend in concentration reduction in our survey of the STP that additionally used ozone treatment (see section 3.1). We compared the results with those reported in a previous assessment of the effectiveness of ozone treatment of dozens of pharmaceutical and personal care products, including antibacterial, antipyretic analgesic, and antihypertensive drugs [46,47]; these other reports also suggested that anti-influenza drugs tend to be easily removed. Although unconfirmed, the reason why the rates of removal of ZAN and LAN by ozone treatment were very similar and slower than those of OS, OC, and PER may be because ZAN and LAN have very similar chemical structures. In this regard, further examination of the relationship between chemical structure and removal efficiency by ozone treatment is important.

The environmental risk posed by anti-influenza drugs in the water environment—namely that their presence may increase the development of drug-resistant viruses in wild ducks [7,48,49]—is especially of concern for OC. The OC exposure concentration needed for the development of Tamiflu-resistant influenza viruses in wild ducks is 1 μg/L [8,9]. In addition, because the pharmacological activity of OS is only about one-hundredth to one-thousandth of that of OC [50,51], even at the time of a large-scale influenza pandemic OS itself probably does not pose an environmental risk [32,52,53]. On the other hand, in the case of ZAN, PER, and LAN there are no available reports on the development of drug-resistant viruses in wild animals. Environmental risk assessments [2,7,48] have been performed on the basis of values in the literature for half-maximal (50%) inhibitory concentrations (IC_50_) against neuraminidase in vitro; these values are used to assay the pharmacological effects of anti-influenza drug components on influenza viruses. The IC_50_ value for OC is about 70–500 ng/L [54–60]; it is about 100–300 ng/L for ZAN, [54–57,59,60], 30–400 ng/L for PER [18,54,57], and 150–700 ng/L for LAN [59,60]. Although the values are somewhat lower for OC than for the others, they are all similar. Comparison of these values with the concentrations we detected in river water at the time of the seasonal influenza outbreak reveals that, even though the components had a broad range of about one-half to one-thirtieth of the IC_50_ values, none had concentrations that could pose an immediate environmental risk and become a problem.

Nevertheless, many studies [32,48,61,62] have pointed out the potential increase in environmental risk due to concentrated administration of anti-influenza drugs, including the risk posed by a large increase in the river concentrations of these drugs in the event of a future worldwide pandemic caused by a new strain of influenza virus. For this reason, it is important to encourage the use of ozone treatment, which can effectively remove a wide range of components, in the sewage treatment process. This can mitigate pollution loads in the river environment, not only from the components of anti-influenza drugs but also from the components of other pharmaceutical and personal care products, thus lessening the risk of drug-resistant viruses developing in the river environment.

## Conclusions

We conducted a detailed monitoring survey of river waters and of STP effluent flowing into urban rivers in the highly populated Yodo River basin, Japan. In addition to OS, OC, and ZAN, the survey covered the new drug components PER and LAN, which were clinically introduced in Japan in 2010.

To our knowledge, this is the first research to confirm the presence of PER and LAN in river water and STP effluent during the winter influenza outbreak season. In addition, we confirmed through STP field experiments and laboratory-scale batch experiments that ozone treatment was effective in removing all five anti-influenza drug components and found the drug dependent difference in susceptibility against ozone. These findings should be valuable for conducting detailed environmental assessments of the pollution caused by the components of anti-influenza drugs in the river environment.
